# Gendered conflict in the human family

**DOI:** 10.1017/ehs.2023.8

**Published:** 2023-04-24

**Authors:** David W. Lawson, Sarah Alami, Oluwaseyi Dolapo Somefun

**Affiliations:** a Department of Anthropology, University of California Santa Barbara, USA; b The School of Collective Intelligence, Mohammed VI Polytechnic University, Morocco; c University of the Western Cape, South Africa

**Keywords:** sexual conflict, cultural evolution, social learning, marriage, reproduction

## Abstract

Sexual conflict is a thriving area of animal behaviour research. Yet parallel research in the evolutionary human sciences remains underdeveloped and has become mired by controversy. In this special collection, we aim to invigorate the study of fitness-relevant conflicts between women and men, advocating for three synergistic research priorities. First, we argue that a commitment to diversity is required to innovate the field, achieve ethical research practice, and foster fruitful dialogue with neighbouring social sciences. Accordingly, we have prioritised issues of diversity as editors, aiming to stimulate new connections and perspectives. Second, we call for greater recognition that human sex/gender roles and accompanying conflict behaviours are both subject to natural selection and culturally determined. This motivates our shift in terminology from sexual to gendered conflict when addressing human behaviour, countering stubborn tendencies to essentialise differences between women and men and directing attention to the role of cultural practices, normative sanctions and social learning in structuring conflict battlegrounds. Finally, we draw attention to contemporary policy concerns, including the wellbeing consequences of marriage practices and the gendered implications of market integration. Focus on these themes, combined with attendance to the dangers of ethnocentrism, promises to inform culturally sensitive interventions promoting gender equality worldwide.

## Graphical abstract

**Figure:**
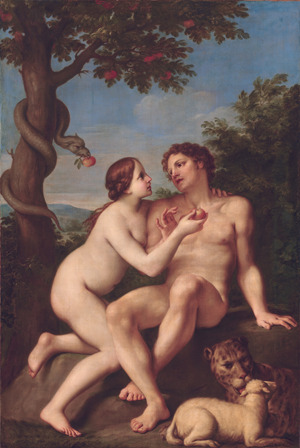

**Social media summary:** Cultural practices structure gendered conflict in human families.

## 1. Introduction

### 1.1. Sexual conflict

For biologists, sexual conflict refers to conflict between the evolutionary interests of females and males, such that the optimal state for one sex imposes fitness costs on the other, often leading to corresponding adaptations and counteradaptations as each sex attempts to gain the upper hand (Parker, 1979, 2006). While early notions of sexual conflict can be found in the writings of Darwin (1871), the concept was not explicitly defined until the behavioural ecology revolution of the 1970s, marking a wider shift away from viewing families as inherently harmonious to being characterised by divergent optima for mating pairs, for parents and offspring, and for siblings competing over parental care and resources (Parker, 1979; Trivers, 1974). These conflicts exist because the fitness implications of alternative phenotypes are frequently distinct for interacting but genetically non-identical individuals. In the case of sexual conflict, there may be differing ideals, for example, about whether two individuals should mate, when they should have offspring and how to care for them. The extent of conflict can be quantified as the difference between female and male optima, what Godfray (1995) refers to as the ‘battleground’, and may only be minimised by rare scenarios of obligate lifetime monogamy, such that the reproductive success of every paired female and male is perfectly aligned. It is also useful to consider the ‘conflict load’, as the amount by which the fitness of either sex is below its hypothetical optimal value for a trait (Lessells, 2006). The outcome, or ‘resolution’, of sexual conflict occurs when each sex has no remaining options to manipulate one another, or when what options do remain have their own fitness costs that balance the anticipated reduction in conflict load (Lessells, 2012).

The study of sexual conflict did not fully take off as a dedicated research area in behavioural ecology until the 1990s (for discussion see: Parker, 2006; Tregenza et al., 2006). Over the last few decades theoretical and empirical investigation has expanded, with sexual conflict now studied extensively across the natural world. For example, in a comprehensive review, Palombit (2014) categorises non-human primate sexual conflict into pre-copulatory strategies such as harassment and forced copulation, and post-copulatory behaviours such as infanticide and mate guarding. This work identifies not only coercive tactics of males, but also female (counter)strategies, including soliciting extrapair matings and behaviours that confuse paternity, such that males misdirect paternal care (see also Stumpf et al., 2011). While these studies often document overt behavioural conflict, it is important to emphasise that sexual conflict applies more broadly to accompanying patterns of selection. Put another way, sexual conflict theory can hold explanatory power in the study female and male relationships even when each sex superficially appears content with the status quo.

### 1.2. Applying the framework to human behaviour

Barbara Smuts, primarily known for her work on non-human animals, was among the first to apply the notion of sexual conflict to human behaviour. In a series of seminal articles, she speculated on the evolutionary origins of patriarchy and the drivers of cultural variability in conflicts between women and men (Smuts, 1992, 1995, see also Hrdy, 1997). Notably, Smuts adopted the terminology of sexual conflict only sparingly, instead referring more generally to patterns of sexual coercion and male aggression to women. A key insight from this pioneering work is that variability in subsistence and residence patterns is highly influential to the expression of conflict. For example, where men control a greater share of resources, such as in transitions from foraging to agriculture, and/or when women are separated from kin by patrilocal post-marital residence, women's ability to counter male coercion may be particularly limited. Furthermore, Smuts recognised the unique impact of culture in humans, an observation she supported by highlighting a long tradition of anthropological scholarship documenting cultural variation in gender ideology. Interpreting such norms as shaped by selection, Smuts hypothesised that our capacity for culture, including language, enables the creation and propagation of ideologies of male dominance and supremacy, allowing men to consolidate their control over women (Smuts, 1992, 1995).

Almost two decades after Smuts, Borgerhoff Mulder and Rauch (2009) reviewed progress in our understanding of conflict between women and men across cultures from an evolutionary perspective, this time more closely following the framework of sexual conflict theory. Here, they highlight research streams addressing divergent optima for marriage practices, including variability in how infidelity and divorce may benefit one sex more than the other, deceptive signalling in mate attraction and differences in ideal family size. For instance, because women typically make greater contributions to parental care than men, and women's desirability on the mating market tends to decrease with age and parity more so than for men, men might be predicted to have a higher optimal number of offspring. Many studies of female vs. male fertility preferences are consistent this notion, but the pattern is far from universal, suggesting important interactions with contextual factors, such as norms about divorce and remarriage that dictate the extent to which wives exhausted by the costs of high fertility may be replaceable. The underlying logic of a hypothesised sexual conflict over family size has since been further questioned by Moya et al. (2016). They conclude that a higher fertility preference for men than women will only be predicted for those men, often in the minority, with sufficiently high mate value to successfully attract sequential mates.

In their review, Borgerhoff Mulder and Rauch (2009) raised two important concerns. First, despite clear indications that sexual conflict is ubiquitous in humans, dedicated evolutionary scholarship remains underdeveloped compared with the flourishing literature on non-human animals. Indeed, many of studies reviewed by Borgerhoff Mulder and Rauch (2009) come from adjacent disciplines like economics (which shares some key assumptions with the behavioural ecological paradigm; Nettle et al., 2013), rather than evolutionary social science *per se*. Here, it might be countered that evolutionary psychologists have a developed a robust study of sexual conflict in human mating strategies (Buss, 2017; see also Perry & Chapman, 2023). Yet research in this subfield has, historically at least, been preoccupied with identifying ostensibly species-typical trends, rather than addressing the abundant variation observed in sexual conflict battlegrounds across cultures so strongly emphasised by Smuts (1992, 1995) and Borgerhoff Mulder and Rauch (2009). Recent psychologically orientated research suggests a trend towards investigating context-dependency in strategies of sexual conflict, albeit with focus primarily remaining on high-income nations. Brooks et al. (2022), for example, report that regional signifiers of high male–male competition for mates within the United States, such as male-biased sex ratios, are associated with a greater prevalence of involuntarily celibate, or ‘incel’, men promoting misogyny online.

The complexity of modelling sexual conflict may itself have stalled research developments since it requires measurement of (or at least informed speculation on) the simultaneous costs and benefits of alternative behavioural phenotypes for more than one individual. Illustrating this point, an extensive literature on mating preferences for age is predicated on the notion that men prefer youth as a cue to reproductive value and women prefer seniority as cue to status or wealth. However, research in this area (as recently reviewed by Conroy-Beam & Buss, 2019) has rarely entertained the possibility of a conflict of interest over the magnitude of age gaps between partners. In contrast, across the social sciences, large age gaps are generally assumed to be both a product and determinant of gender inequality, with male seniority encouraging women's subordination i.e. large husband-older age gaps benefit men at a cost to women (e.g. Carmichael, 2011). Studies examining the fitness battleground of spousal age gaps remain surprisingly rare, and have produced mixed results (see Lawson et al., 2021a; Minocher & Ross, 2022). Additional complexity in modelling sexual conflict is introduced by recognising that kin also have an unusually strong influence on mating arrangements among humans (Borgerhoff Mulder & Rauch 2009). As a consequence, strategic interests and behaviours of parents and in-laws, not just women and men, need to be considered, such as in Baraka et al.'s (2022) analysis of the costs and benefits of early marriage for women, their spouses and their parents.

Borgerhoff Mulder and Rauch's (2009) second criticism is that recent theoretical developments in sexual selection research originating in the non-human animal literature have been slow to permeate to parallel human-focused research (see also Borgerhoff Mulder, in press). Conventional applications of sexual conflict theory emphasise the significance of sex differences in potential reproductive rates, with females committed to larger minimal prezygotic and postzygotic investments per offspring, such that males have more to gain from maximising mating opportunities, while females have more to gain from more selective mating and continued resource allocation to parental care (Clutton-Brock & Vincent, 1991; Trivers, 1972). This framework, referred to as the ‘Darwin–Bateman paradigm’ (Dewsbury, 2005) following Bateman's (1948) study concluding that males alone are under selection to pursue multiple mating partners in *Drosophila*, has been subject to intense debate in recent years (reviewed in Hoquet, 2020; Morimoto, 2020; Rosenthal & Ryan, 2022).

Questions have been raised about the validity of Bateman's original data (Hoquet et al., 2020), failures to replicate his findings (Gowaty et al., 2012) and their wider applicability across the animal kingdom (Fromonteil et al., 2023; Janicke et al., 2016; Kokko & Jennions, 2023). Moreover, alternative ecological factors, such as sex-specific patterns of mortality, population density and the local sex ratio, have been demonstrated to be important in determining sexual selection on female and male optima (Kappeler et al., 2022; Kokko & Jennions, 2008; Kokko & Monaghan, 2001). For example, if the sex ratio is male biased, then selection may favour males striving to obtain and maintain access to a singular mate, rather than competing for additional partners (Kokko & Jennions, 2008). Among humans, despite a large corpus of demographic studies, our evidence base for assessing variability in female and male mating and reproductive success remains surprisingly limited (Borgerhoff Mulder, in press; Brown et al., 2009). What data we do have indicate that patterns of selection on competing for, or choosing, mates are far from universal. In particular, the accompanying costs and benefits of multiple mating probably vary in tandem with the cultural acceptability of polygamy, divorce and remarriage, with scope for differential selection by sex limited to those societies characterised by polygyny or serial monogamy and minimal when lifetime monogamy is the norm (Brown et al., 2009).

The relatively small literature on sexual conflict in humans, and its sometimes stubborn adherence to classic, but limited, theoretical models of conflict, arguably reflects a wider tendency of researchers to draw primarily from pioneering research up to the late 1970s, and for human and non-human focused research to develop semi-independently since then, a problem West et al. (2011) refers to as the ‘*disco problem*’ (see also Nettle et al., 2013). Likewise, Perry and Chapman (2023) stress a continuing lack of dialogue across research fields dealing with different taxons, and a wariness of researchers working on non-humans to tackle the complexity of integrating culture into a sexual conflict perspective. Fortunately, however, there is every indication that updated models of sexual selection and sexual conflict are increasingly being applied to humans. Here, for example, we can refer to recent studies examining the payoffs to multiple mating for women (Borgerhoff Mulder, 2009; Scelza, 2013), the dynamics of extra-pair mating and reproduction (Scelza et al., 2020), and the influence of sex ratios on mating and parenting strategies (Schacht & Borgerhoff Mulder, 2015; Uggla & Mace, 2017) and on rates of violent conflict (Schacht et al., 2014). We hope this special collection further stimulates this effort, while also highlighting the potential benefits of improved dialogue with non-evolutionary social scientists studying conflict between women and men.

### 1.3. Navigating continuing controversy

It is also important to recognise that evolutionary studies of sex and gender have been mired in controversy, perhaps leading some researchers wary to wade into dangerous waters. Infamously, Thornhill and Palmer (2000) drew considerable fire for their book *A natural history of rape*, exploring the idea of rape as an adaptation. A full review of their arguments is beyond our scope, but it is perhaps their encouragement of a false dichotomy between feminist and evolutionary perspectives on intimate partner violence (IPV) that is largely responsible for their legacy of doing more to burn, rather than build, bridges with social scientists working on related themes. Vandermassen (2011) provides a particularly balanced perspective on this controversy, describing the book as a missed opportunity to integrate perspectives, while also arguing that synergistic opportunities will only be gained if more feminists relinquish blanket hostility to evolutionary thinking. Reviewing matters more broadly, Liesen (2007) differentiates between the work of behavioural ecologists and evolutionary psychologists, arguing that the latter have exhibited a consistent ‘chill’ towards feminism (see also Fausto-Sterling et al., 1997). In contrast, human (and non-human primate) behavioural ecologists, via a greater focus on contingency and adaptive plasticity, have done more to explore contextual influences on female–male relationships, effectively opening up opportunities to correct old biases and explore social determinants of behaviour typically emphasised in feminist scholarship.

Subdisciplinary boundaries are of course fuzzy (Sear et al., 2007), and this is not the place to review the history and differing assumptions of evolutionary psychology, behavioural ecology and approaches to cultural evolution (see Laland & Brown, 2011; Smith et al., 2001). Our point here is only to emphasise that a variety of beliefs about the meanings and implications of sex and gender are compatible with an evolutionary perspective, including extensions beyond the sex/gender binary (DuBois & Shattuck-Heidorn, 2021; McLaughlin et al., 2023). Moreover, evolutionary scholarship that is informed by, and contributes to, feminist thought is fully realisable, especially when socioecological contingencies are fully explored. Illustrating this point, Darwin's early writings, while clearly infused by harmful Victorian gender stereotypes (Fuentes, 2021a; Rosenthal & Ryan, 2022), were heralded by some of his contemporaries as serving a feminist agenda because human nature was presented, not as a fixed constant, but rather as open to change and environmental influence. In contrast to the prevailing ideas of the time, an evolutionary perspective was therefore interpreted as showing there is in fact nothing permanent or incontrovertible about women's subordinate place in society (Brilmyer, 2017).

Opportunities and best intentions aside, evolutionary studies of sex and gender run a risk of reinforcing harmful gender stereotypes. A typical response to this dilemma is to point to common misunderstandings of the relationship between evolution and behaviour, such as naive portrayals of evolutionary social science as genetic determinism, or false assumptions that humans are predicted to consciously strategise about fitness (see for example, Gibson & Lawson, 2015). While such misunderstandings present an important roadblock, a more proactive stance requires reflecting and acting upon, often implicit, biases in how evolutionary scholarship is conducted and presented (Ah-King, 2022; Karlsson Green & Madjidian, 2011). Ever since *The women that never evolved* (Hrdy, 1981), Sarah Hrdy has been especially instrumental here in highlighting the myriad ways in which gender stereotypes have been indulged and women's evolved strategies have been overlooked by a largely male academy (see also Hrdy, 1999). Decades later, we are still addressing these same issues, and evolutionary studies of sex and gender remain steeped in controversy (Ah-King, 2022; Cooke, 2022; Sani, 2017).

Burch (2020), for example, concludes that evolutionary psychology textbooks routinely discuss the dynamics of female physical attractiveness, while saying little about women's intelligence and resourcefulness, and overemphasising the role of men in provisioning their families. On this same theme, Sear (2021) tackles a widespread myth that a male breadwinner–female homemaker nuclear family is the ‘traditional’ family structure, arguing that cross-cultural and historical observation reveals remarkable flexibility in childcare responsibilities and the division of labour (see also Bliege Bird & Codding, 2015; Starkweather et al., 2020). A recent special issue is devoted to women's cooperative relationships, arguing that their importance in the evolution of human sociality has, until recently, been systematically neglected (Fox et al., 2023). Another is devoted to critiquing the idea of any single ‘natural’ form of masculinity (Gutmann et al., 2021). Here, Fuentes (2021b) argues that pervasive assumptions about the ancient roots of gender differences are poorly supported by contemporary scholarship on primate behaviour and hominin prehistory. Nelson (2021) further proposes that evolutionary anthropologists have to date placed an outsized focus on physical forms of violence (and particularly male-male conflict), leaving questions of structural violence (i.e. the creation of and maintenance of discriminatory and exclusionary social and institutional structures) relatively undertheorised. While it might be countered that evolutionary anthropologists have long studied political and economic inequality, particularly with respect to shifts in subsistence mode (Mattison et al., 2016), we agree that further dedicated research into the (cultural) evolution of relevant complex institutions would be desirable (see also Currie et al., 2021), including their implications for gender differences in social status (see also Smith et al., 2021).

It is clear that we are at an important juncture. We still have much to learn about conflict between women and men, yet we must also tread carefully to avoid past missteps and biases, and misunderstandings and misapplications of our scholarship. To this end, we advocate for three synergetic research priorities, including a strategic change in terminology from *sexual conflict* to *gendered conflict*. In the next sections, we lay out our rationale and supporting arguments for each of these priorities, while weaving in observations from the papers included in this special collection. We then end with some final thoughts on the future of sexual/gendered conflict research.

## 2. Three priorities for future research

### 2.1. Prioritising diversity

Our first proposition is a concerted commitment to diversifying perspectives and methods in order to innovate the field, achieve ethical research practice and foster more fruitful dialogue with neighbouring social sciences. It is now widely accepted that improving the representation of minoritised and underrepresented groups is not only essential from a social justice perspective, but also leads to critical intellectual shifts that improve scholarship (AlShebli et al., 2018; Bolnick et al., 2019). As we have highlighted above, the increased representation of women in evolutionary social science illustrates this point well, with women continuing to lead the charge in tackling gender stereotypes and studying women's adaptive strategies (e.g. Borgerhoff Mulder, in press; Fox et al., 2023; Mace, 2013; Scelza, 2013; Sear, 2021). Appropriately, this volume is dominated by women authors.

While barriers to gender equality in science remain (e.g. Fox et al., 2019; Heidt, 2023), we must be attentive to inclusion of other underrepresented groups. Here we turn our focus to achieving representation from varied cultural backgrounds, particularly from scholars from outside of Europe, North America and other relatively high-income regions (see also Mughogho et al., 2023; Urassa et al., 2021). This is critical for several reasons. First, there can only be limitations from excluding researchers with a wider array of life experiences. Not least, it limits our collective capacity to avoid ethnocentric bias. Second, while low and middle-income countries (LMICs) by no means have a monopoly on gender inequality, they also experience the greatest disparities in health, education and apparent bargaining power between women and men (Jayachandran, 2015). Gender inequality is now a major focus of global health research and policy. It is only appropriate then that LMIC scholars are represented. Finally, in both global health (The Lancet Global Health, 2018) and cross-cultural social science, including anthropology, psychology and economics (Urassa et al., 2021), ‘parachute’ and ‘parasitic’ research practices remain commonplace. Too often, research is conducted in LMICs by visiting researchers (i.e. of a different nationality/cultural background to the study population) without collaboration with local communities, researchers and research institutions, or collaboration occurs but is undervalued and uncredited.

In an effort to address these issues, we purposely sought out contributions led by authors from LMICs (Akurugu et al., 2022; Baraka et al., 2022; He et al., 2022), or where research is conducted in LMICs in collaboration and co-authorship with research institutions and/or scholars of the same nationality/cultural background as the study participants (Agey et al., 2023; Mattison et al., 2023; Schaffnit et al., 2023). We also asked all contributors to consider the criteria they used for authorship decisions, referring them to Morton et al.'s (2022) guidelines on promoting equitable authorship (including avoidance of ‘token’ authorship). To minimise bias in the review process, we assigned papers to at least one peer reviewer from the same country or world region as the population under study. Nevertheless, inequalities remain apparent. Most notably, in only one paper using data from an LMIC are all authors of the same nationality as the population studied (Akurugu et al., 2022). Diversity was also lost across the development of the special collection; several contributions were rejected after peer review or because invited authors ultimately opted to submit their manuscript elsewhere. These dynamics surely attest to the barriers faced by LMIC scholars and limited incentives for engaging with a field that may not presently share the same priorities. To round out the collection, we then added several previously published papers addressing sexual/gendered conflict (Kerry et al., 2021; Lawson et al., 2021b; Snopkowski & Nelson, 2021; Starkweather et al., 2020).

Akurugu et al. (2022) exemplify the value of diversifying perspectives in their article on bridewealth, i.e. a transfer of capital from the groom's to the bride's family. Bridewealth is typically framed as harmful to women, for which there is solid evidence. For example, using a vignette experiment wherein the completeness of bridewealth payments is manipulated, Horne et al. (2013) convincingly document a link between bridewealth and normative constraints on women's reproductive autonomy. Reviewing the wider literature, Akurugu and colleagues counter that the ‘foreign gaze’ (see Abimbola, 2019) of most scholarship on bridewealth is characterised by unsettling generalisations and stereotypes, such as labelling bridewealth-practising groups as ‘primitive’ or assuming *a priori* that bridewealth is inherently harmful to women. Drawing on their ethnographic work in Ghana (Akurugu et al., 2021), they counter that, when considered as part of wider patriarchal context that limits women's empowerment more broadly, bridewealth serves a critical purpose in legitimising relationships and the associated rights of women and children from the marriage. As such, women frequently attest support for bridewealth and abolishing the practice may only worsen their status. Akurugu et al. (2022) also make the novel contribution of suggesting that conflicts of interest over women's autonomy may be best settled by empowering overlooked indigenous systems, such as the use of traditional courts to address marital disputes.

We also showcase methodological diversity. Several papers utilise qualitative approaches (Agey et al., 2023; Akurugu et al., 2022; Baraka et al., 2022), rarely at the forefront of contemporary evolutionary social science. Baraka et al. (2022) demonstrate the value of mixed methods research in their study of Tanzanian marriages. Global health frameworks characterise marriages under 18 years as harmful ‘child marriages' serving the interests of parents (e.g. through a larger bridewealth or smaller dowry), and/or husbands who prefer younger wives, while brides pay only costs (e.g. greater risk of pregnancy complications, IPV and school dropout). In earlier work by the same team, testing these assumptions using quantitative data on partner preferences, bridewealth and women's wellbeing and reproductive success led to only limited support (Lawson et al., 2021a; Schaffnit et al., 2019). In particular, marriage under 18 years held mixed, rather than purely negative, relationships with the well-being of adolescent girls and young women, and was associated with higher reproductive success. Baraka et al. (2022), through analysis of focus groups and in-depth interviews, also reject parent–offspring conflict as a primary driving force behind early marriage. Community members believed that remaining unmarried did not shield adolescent girls from risky sexual behaviour and that early marriage often provides relative social and economic security. However, support for gendered conflict was stronger; some adolescent girls were described as being lured into unstable early marriages by men misrepresenting their long-term intentions. This marital scenario may have been previously overlooked because (a) such ‘trick’ marriages only represent a fraction of marriages in the community, such that their costs are masked when analysed alongside relatively neutral or advantageous marital scenarios, and (b) naive assumptions that a gendered conflict model requires active coercion. Here, adolescent girls entered marriages willingly, sometimes against parental wishes, but later came to regret the decision when the marriage failed to meet their expectations.

Anderson and Bidner (2022) further address what may be gained by embracing the overlapping concerns of economics and evolutionary social science, taking polygynous marriage as a case study. Indeed, it is striking how much recent scholarship in economics addresses cultural variation in conflict between women and men, often drawing heavily on classic anthropological scholarship for theoretical inspiration. For example, recent papers address the role of not only marriage practices (Anderson & Bidner, 2022), but also contemporary and historical modes of subsistence (e.g. Alesina et al., 2013; Becker, 2019; Hansen et al., 2015) and kinship systems (Lowes, 2020) in determining gender inequality. As we argue below, there is also much to gain from boosting engagement with neighbouring research traditions, both non-evolutionary and evolutionary, that emphasise the role of culture in determining behavioural diversity.

### 2.2. Emphasis on culture

Our second proposition is a more forceful recognition of the role of culture in the expression of human sex/gender roles and accompanying conflict between women and men. This call is not novel, with the importance of integrating cultural forces into evolutionary models emphasised by Smuts (1992, 1995), Borgerhoff Mulder and Rauch (2009) and many others (e.g. Wood & Eagly, 2012). However, we propose that scholarship needs to be much more intentional in addressing the role of inherited cultural practices, normative sanctions and evolved social learning mechanisms in structuring conflict battlegrounds and conflict resolution. To encourage this priority, we propose a strategic shift in terminology from ‘*sexual conflict*’ to ‘*gendered conflict*’ when addressing human behaviour. This corresponds to the common usage of ‘*sex*’ as emphasising differing chromosomes, and external genitals, which typically serve as the basis for sex assignment at birth, and ‘*gender*’ as emphasising societal norms and expectations of behaviour, and personal identification (for a discussion of alternative definitions of sex and gender, and their limitations, see Hyde et al., 2019; Muehlenhard & Peterson, 2011). An emphasis on culture does not negate that conflict at an evolutionary level ultimately plays out via differential selection on biological sexes. However, emphasising gender over sex places appropriate focus on how the behaviour of women and men is *also* fundamentally socially acquired and transmitted.

More than steer the direction of research, our proposed change in terminology reinforces two foundational points, particularly with respect to how our research is (mis)understood by researchers working outside of the evolutionary human sciences. First, by embracing the term gender, and its connotation with social and cultural influences, we make immediately clear that such influences do not sit outside of the scope of an evolutionary perspective. Likewise, we steer folks away from the stubborn assumption, associated with the connotations of ‘sex’, that an evolutionary perspective dictates that differences between women and men can, or should, be essentialised to chromosomes, hormones or other ‘biological’ essences (for discussions of the false nature-nurture, biological–cultural/social dichotomy see Eagly & Wood, 2013; Nettle, 2009, 2018). Second, adopting a distinct terminology for humans and nonhuman behaviour, we reinforce acknowledgement of fundamental differences between these taxonomic groupings, particularly our propensity for complex cumulative culture (Mesoudi & Thornton, 2018). Making this distinction explicit will hopefully instil appropriate caution in researchers when applying theory based on non-human animals to humans.

To draw greater attention to cultural forces, Table 1 highlights findings from recent and classic scholarship on a range of relevant practices that structure gendered conflict, including articles from this collection and research carried out by scholars working outside of evolutionary social science. Each practice can be considered cultural because associated behaviours are acquired socially, such that women and men's behaviour in large part reflects a matter of tradition rather than preferences required by individual learning, and the costs and benefits of associated behaviours are modified by normative sanctions, i.e. rewards for compliance and/or punishments for deviation. Note that here we have purposely highlighted potentially contrary findings that could be interpreted as indicating that a practice is, or is not, a site of gendered conflict (and so harmful to one gender or both). In many cases, the impacts on women and men, and the relevant selective forces at stake, remain subject to debate.

**Table 1.** Cultural practices structure gendered conflict. Conflict between women and men is influenced by cultural context; relevant behaviours are acquired socially and influenced by normative sanctions. However, there remains much debate about the extent to which certain cultural practices impact fitness and wellbeing. Here, we highlight examples of, sometimes contrasting, findings across the literature on the potential costs and/or benefits of a range of cultural practices, including work by both evolutionary and non-evolutionary social scientists. These examples are not intended to be fully representative, but rather illustrative of the diversity of existing scholarship.

Practice	Example finding(s)	Context	Reference
Arranged or forced marriage	Parents and daughters more often disagree than parents and sons over desired qualities in prospective marriage partners, and marriages with no parental consent or input bear harsher consequences for women than men.	Dhading, Nepal	Agey et al. (2023)
	Parents and daughters more often disagree than parents and sons over desired qualities in prospective marriage partners.	Yunnan, China	Bovet et al., (2018)
Early marriage	Men lure adolescent girls/young women into unstable marriages with false promise of providing social/economic security.	Sukuma, Tanzania	Baraka et al. (2022)
	In contexts where returns to education are low and economic opportunities are limited for young women, early marriage is a desirable option for girls and young women that leads to greater autonomy from parents and higher status within the community.	Sukuma, Tanzania	Schaffnit et al. (2021)
Polygynous marriage	The size and composition of polygynous households is associated with greater livelihood resilience compared with monogamous households.	Mali (multiple regions)	Dessy et al. (2021)
	Polygynous marriage is associated with a greater degree of emotional distress for women compared to monogamous marriage.	Aleppo, Syria	Maziak et al. (2002)
	Children in polygynous families have a higher risk of early death and a slower rate of growth than children in monogamous families.	Dogon, Mali	Strassmann (2011)
	Children in male-headed polygynous households (typically first wife households) have equal or better health than children in male-headed monogamous households.	Northern Tanzania (multiple regions)	Lawson et al. (2015)
Large spousal age gaps	Women in relationships with men more than 15 years older than themselves are at an increased risk of experiencing spousal violence.	Nigeria, Tanzania (multiple regions)	Izugbara (2018)
	Women married to relatively younger men suffer increased risk of spousal violence.	Sukuma, Tanzania	Kilgallen et al. (2022)
Female genital mutilation/cutting (FGMC)	Cut women have more surviving offspring in regions where FGMC is widespread.	Mali, Nigeria, Senegal, Ivory Coast, Burkina Faso (multiple regions)	Howard and Gibson (2017)
	Cut women report more restricted sociosexually than women without FGMC.	Igbo, Nigeria	Onyishi et al. (2016)
	FGMC is not associated with women's self-reported engagement in pre-marital sex.	Mali, Nigeria, Senegal, Ivory Coast, Burkina Faso (multiple regions)	Howard and Gibson (2019)
Intimate partner violence (IPV)	Patterns of IPV suggest that men resort to violence to limit women's reproductive autonomy and to coerce them into producing more children than they desire.	Tsimane, Bolivia	Stieglitz et al. (2018)
	Men use IPV to control their wives’ responses to their infidelity and the subsequent diversion of resources away from the family.	Tsimane, Bolivia	Stieglitz et al. (2011)
	Indicators of paternity concern and paternal disinvestment are associated with an increased risk of IPV.	Burkina Faso, Chad, Ethiopia, Gambia, Ghana, Ivory Coast, Kenya, Malawi, Mali, Nigeria, Togo, Zambia	Howard and Gibson (2023)
Bridewealth	Bridewealth payments strengthen normative constraints on women's reproductive autonomy.	Volta region, Ghana	Horne et al. (2013)
	Women concurrently value gender equality and the practice of bridewealth. They associate men's payment of bridewealth with their willingness to support women's autonomy and intimate relationships.	KwaZulu–Natal, South Africa	Yarbrough (2022)
Dowry	Overall, dowry payments are associated with a greater risk of IPV. However, the association between dowry payments and IPV risk is inverse V-shaped. Women in marriages where no dowry or very large dowries were paid experience a lower risk of violence.	Chapainawabganj, Chittagong and Sherpur districts, Bangladesh	Suran et al. (2004)
	Dowry payments are associated with greater standard of living for women and help to ensure high levels of investment in her future offspring by her spouse and in-laws.	Bangalore, India	Shenk (2007)
Menstrual taboos	Menstrual taboos and the use of menstrual huts, which restrict women's freedom of movement, are associated with greater paternity certainty.	Dogon, Mali	Strassmann, (1992), Strassmann et al. (2012)
Post-marital residence	Women have a heavier workload in patrilocal compared with matrilocal communities, where men may benefit from their higher bargaining power to do less work.	Mosuo, Han and Yi populations of Southwest China	Chen et al. (2023)
	Within matrilocal settings, men are less likely to help on their wife's farm when there are more women reproducing in the wife's household.	Mosuo, southwest China	He et al. (2022)
Witchcraft accusations	Husbands accuse their wives of witchcraft if they suspect them to be unfaithful or to gain greater control within the marriage.	Bantu societies in central and southern Africa	Peacey et al. (2022)
	Women resort to witchcraft to level intra-household disparities.	Meru district, Kenya	Dolan (2002)
Restrictions on women's clothing	In harsh environments that beget high levels of paternal investment, men are relatively more supportive of veiling than women.	26 nations where restrictive dress is practised.	Pazhoohi and Kingstone (2020)
	Wearing the hijab is perceived as a symbol of identity and empowerment by women.	Muslim women in France	Croucher (2008)
Widow inheritance/levirate marriage	Automatic marriage of a widow to a patrilineal relative may provide social protection when women lack property rights or means of economic support.	Tanzania	Kudo (2021)
	Widow inheritance is associated with cleansing rituals which pose a risk of transmitting HIV.	Nyanza Province, Kenya	Perry et al. (2014)

In considering these practices, one important goal then is to interrogate alternative hypotheses about who, if anyone, gains (in an ultimate and proximate sense) from relevant practices and how. Howard and Gibson (2023), for example, test hypotheses that IPV, which varies widely in prevalence and has a strong normative component, reflects a male strategy to (a) reduce perceived threats to their paternity certainty, (b) impose a higher fertility optimum than their partners via coercive sexual activity and/or (c) to quell spousal objections to diverting resources outside of the family (see also Stieglitz et al., 2011). Utilising proxies for anticipated levels of paternity certainty, contrasting fertility preferences and extra-marital relationships in African national survey data, they conclude that patterns are most consistent with paternity uncertainty risk and paternal disinvestment hypotheses. They also highlight limitations to their analysis, including ambiguity in causal relationships between covariates included in their models, stifling capacity for causal inference. We suggest that future research must also recognise that an absence of IPV need not equate with a lack of conflict. Indeed, women who do not challenge male dominance (e.g. never oppose men's investments outside of marriage) because of an anticipated threat of IPV or other harmful consequences may suffer the largest conflict load, with IPV incidence itself consequently more reflective of situations were women have sufficient bargaining power to make challenging male authority a worthwhile risk (see Kilgallen et al., 2022).

A second overarching goal is to understand why relevant cultural practices vary across time and space. This can be considered an exercise in elucidating the ‘*evolutionary and ecological roots*’ of gender inequality (see also Kaplan et al., 2009), as we work to identify legacies of selection and adaptation in response to interrelated socioecological factors such as mode of subsistence, mortality risks, sex ratio, population density and intergroup relationships. It is important to note here that emphasising socioecological contingency in this way does not equate with assuming genetic adaptation to particular socioecological conditions, but rather envisages humans as bestowed with a remarkable capacity for adaptive phenotypic plasticity (Nettle et al., 2013). Indeed, a one-to-one matching between any single socioecological factor and appropriate behavioural response is unlikely, with adaptive strategies depending on the overall constellation of factors defining a context. Further complicating matters, once a cultural practice becomes entrenched, it may further influence patterns of selection on other behaviours.

Scelza et al. (2021) expertly illustrates this complexity with respect to pastoralism, which typically requires long periods of spousal separation with men transporting livestock to feed away from home. Such absences may ultimately favour social norms that limit women's freedom, so as to ensure confidence of paternity. Supporting this notion, Becker (2019) has documented that women from historically pastoral societies are more likely to have undergone the most invasive forms of female genital mutilation/cutting (FGMC) and adhere to restrictive norms about women's sexual freedom and mobility. However, there are exceptions to this pattern, such as the Himba of Nambia, who combine pastoralism with strong norms promoting women's sexual autonomy. Scelza et al. (2021) speculate that a combination of factors explains Himba exceptionalism, including a history of matrilineal inheritance predating pastoralism, bestowing the Himba with norms favouring sexual freedom for both genders, and both a high reliance on children's labour and a female-biased adult sex ratio, which ultimately allows men to more easily compensate for paternity loss through pursuit of their own extra-marital partnerships.

Modelling the role of cultural history, such as the legacy of matriliny in the above example, presents a particular challenge because it requires more information than can be gained from observing a population at any single time point. Indeed, this challenge applies broadly to optimality models of human behaviour (Barrett & Stulp, 2013). One tool at our disposal is phylogenetic analysis, which can be used to examine how transitions from one cultural state predicts changes in others (Mace & Jordan, 2011). Work in this tradition, for example, has addressed origins and patterns of change in the practice both female and male genital mutilation/cutting (Šaffa et al., 2022), polygynous marriage (Minocher et al., 2019), the direction of marriage payments (Fortunato et al., 2006) and traditions of sex-biased dispersal, i.e. post-marital residence (Ji et al., 2022; Jordan et al., 2009). More generally, these considerations make clear that a robust evolutionary approach to human diversity requires investing in a bedrock of historical and ethnographic knowledge.

At the proximate level, there is also still much to learn about the role of evolved mechanisms of social learning in determining gender ideology (i.e. expectations about appropriate behaviour for each sex/gender). Here, the paucity of evolutionary research is jarring, not least because the notion of socially acquired and ‘performed’ gender roles has motivated a large body of scholarship in sociology and social psychology (Butler, 1988; Morgenroth & Ryan, 2018). Instead, evolutionary scholars studying social learning strategies have devoted their attention to alternative behavioural domains, most obviously cooperative tendencies (Henrich & Muthukrishna, 2021) and, to a lesser extent, reproductive scheduling (Colleran, 2016). Outside of evolutionary social science, many studies convincingly demonstrate a pivotal role of social learning in gendered conflict. Bursztyn et al. (2020), for instance, demonstrate the power of conformity bias: they find that Saudi men underestimate peer support for women's empowerment and that correcting these misperceptions leads to shifts in gender relations, e.g. men becoming more supportive of their wives working outside the home. Swindle (n.d.) addresses pathways of cultural diffusion in Malawi, linking exposure to journalism critically covering IPV with the probability that surveyed men condemn violence. Cano and Hofmeister (2023) consider the vertical transmission of gender norms, demonstrating that, even when controlling for potential confounders, observation of paternal involvement in domestic labour is predictive of Australian adolescents later adopting more equalitarian gender ideology. These examples also lead into our final proposition: evolutionary studies of gendered conflict have much untapped potential to contribute to areas of contemporary policy concern.

### 2.3. Tackling areas of policy concern

There is much scope for evolutionary perspectives not only to contribute to our understanding of patriarchy, but also to provide fresh insights into how gendered conflict and its harmful impacts may be reduced worldwide. As such, our third proposition is that we target topics of contemporary policy concern. There are notable synergies with our first two priorities here. Committing to diversity guards against ethnocentric bias, increasing the likelihood of generating culturally sensitive policy recommendations. Elucidating the role of culture also offers new possibilities for engagement with global health professionals working on gender inequality, whose attention focuses increasingly on tackling inequitable social norms (Jayachandran, 2020). Indeed, the now popular ‘social norms approach’ to behaviour change interventions (see Bicchieri & Mercier, 2014) has much inherent overlap with contemporary approaches to cultural evolution. Most obviously, both share assumptions that behaviour is influenced by perceptions of what others do and believe, and the anticipated rewards or punishments for conforming to, or deviating from, prevailing norms (Eriksson et al., 2021; Kendal et al., 2018).

Several relevant themes emerge in the special collection. The first is the wellbeing consequences of so-called ‘harmful cultural practices’. Since the 1990s, the United Nations and other international development agencies have identified cultural practices as a fundamental determinant of gender inequality, with particular focus on traditions of son preference, IPV, FGMC, polygynous marriage, ‘child marriage’ (<18 years), forced or arranged marriage, bride caputre/kidnapping, bridewealth and dowry (Longman & Bradley, 2016), but also less well-known practices such as ‘breast ironing’ (Amahazion, 2021), and ‘widow cleansing’ (Perry et al., 2014; Manala 2015). However, as several papers in this special collection make clear (see also Table 1), assumptions about the inherent harms of these practices are commonly made with little reference to evidence, opening up much scope for ethnocentric bias (see also Lawson & Gibson, in press). In cases such as child marriage (Baraka et al., 2022), bridewealth (Akurugu et al., 2022), arranged marriage (Agey et al., 2023) and polygynous marriage (Anderson & Bidner, 2022; Lawson & Gibson, 2018; Pesando, 2021), careful analyses present a more nuanced picture of the wellbeing (and fitness) implications of each practice. For example, some studies show that polygynous marriage is predictive of relatively poor health for women and their children, implicating resource competition and co-wife conflict (Omariba & Boyle, 2007; Strassmann, 2011), while others highlight apparent benefits for women, including greater access to male owned wealth and associated benefits of greater livelihood resilience which benefits all family members (Dessy et al., 2021; Lawson et al., 2015).

An evidence-based and culturally sensitive approach to behaviours and practices which appear harmful can lead us away from parochial interventions that punish families making difficult decisions with limited choices. For example, Schaffnit et al. (2021) argue that criminalising marriage under 18 years may be damaging for adolescent girls and young women by constraining their options, *unless* such interventions are also effectively combined with policies addressing the vulnerabilities experienced by those delaying marriage, i.e. exposure to risky sexual behaviour, premarital childbearing and negative social judgements of unmarried women. By adding ethnographically grounded and contextually specific analyses, evolutionary social scientists have much to contribute to the difficult task of disentangling to what extent alternative cultural practices are best understood as a root cause of gender inequality or rather a product of constrained options (or both). Women, for example, may accept the apparent costs of bridewealth on their agency, because to fail to do so risks leaving their children illegitimate, but also because a potential spouse's ability to pay bridewealth is an honest signal of his ultimate ability to provide for her and her future family in contexts where women's ability to generate wealth independently is limited by wider patriarchal norms (Akurugu et al., 2022).

FGMC provides a particularly interesting case where many evolutionary scientists and global health professionals share similar assumptions about the likely motivations behind the procedure, but where attempts to empirically test these assumptions have revealed mixed results. For example, via analysis of African demographic survey data, Howard and Gibson (2017) present data consistent with the importance of FGMC in marriage markets; cut women achieve higher reproductive success than uncut women in areas where the practice is most common. However, in subsequent analyses they also reject the idea that the practice benefits men (from a fitness perspective) by controlling women's sexuality, reporting no association with FGCM and women's reported sexual activity (Howard & Gibson, 2019). Efferson et al. (2015) also piqued the attention of global health professionals by challenging a popular notion that the maintenance of FGMC in a population depends on a critical number of families who cut their daughters and demand cut daughters-in-law for their sons (see Mackie & LeJeune, 2009). Efferson et al. (2015) found no evidence that cutting was coordinated within a large sample of Sudanese communities, and substantial variation in attitudes and cutting behaviour between individual families. This finding implies that rather than allocating limited resources to mass abandonment ceremonies to force numbers below a tipping-point, any intervention which reduces even small numbers of cutters could contribute to a cumulative reduction in FGMC over time (for further discussion see Lawson & Gibson, in press).

A second emergent theme is presented by several papers in the collection addressing how patterns of gendered conflict are being influenced by market integration (Agey et al., 2023; Mattison et al., 2023; Schaffnit et al., 2023). Mattison et al. (2023), for example, incorporate a consideration of matrilineal vs. patrilineal kinship to provide a fresh perspective on long-running debates about the impact of market integration on wealth inequality. They find matrilineal Mosuo in Southwestern China have greater wealth inequality than their patrilineal counterparts, which they attribute to higher levels of market integration among this group. However, within matrilineal communities, greater wealth was associated with lower inequality, highlighting the importance of sharing norms and institutions in counterbalancing inequalities that may otherwise arise. Given the absence of similar redistributive mechanisms in patrilineal groups, they speculate that wealth differences will become relatively more apparent with greater economic development.

Schaffnit et al. (2023) and Agey et al. (2023) address the influence of market integration on patterns of arranged marriage, a site of both potential parent–offspring conflict and gendered conflict (as arranged marriage more often involves coercion of wives and daughters than husbands and sons). Drawing on data from Bangladesh, Schaffnit et al. (2023) find that contrary to their expectations, markers of family market integration do not predict whether women enter an arranged or love marriage. They argue that while access to education and participation in the workforce has opened up more avenues for women to choose their spouse, the adoption of love marriage as an individual practice does not depend solely on socioeconomic factors. Further, they show that dowry payments and gifts continue to be made by parents even when their daughters are the ones who choose their spouse, suggesting parental approval remains an important factor. Agey et al. (2023) highlight the greater potential for disagreement over spouse choice between parents and daughters (relative to sons) in Nepal, where love marriages are on the rise among the younger generation. Parents can choose to withhold dowry payments if they disapprove of the marriage, which can strain daughters’ relationship with her new in-laws. Women who elope against their parents’ wishes may find themselves socially isolated and at greater risk of domestic abuse. In contrast, men are typically less beholden to their parents and so generally receive less scrutiny about their marital choices. These findings highlight the disparate effects of market integration for women and men, which should be considered by policymakers working in societies experiencing rapid socioeconomic transitions.

Finally, several papers address intracultural variation in gender ideology across time and space. Lawson et al. (2021b) consider women's empowerment from a conflict perspective, exploring the correlates of men's gender ideology in a semi-urban community in Mwanza, Tanzania. They find that men's support for women's empowerment is domain specific, and greatest for domains that do not entail an explicit cost to men. For instance, men were largely in favour of the education of girls and women's participation in the workforce and the community's political life, which were perceived to improve both men and women's socioeconomic standing and quality of life. However, they were far less supportive of women's authority over the household decision-making process and were more likely to agree that husbands have a right to engage in IPV. They also find little evidence that potential demographic indicators of gendered conflict (polygynous marriage, large spousal age gap, high fertility) were predictive of men's beliefs, underscoring the notion that these behaviours have more nuanced relationships with gender relations within communities than often assumed by global health professionals, or implied by crude cross-national analyses.

Kerry et al. (2021) and He et al. (2022) also provide novel investigations into the role of kin in determining gender relations. Using data from an online survey in the United States, Kerry et al. (2021) find those who have more male than female kin hold less favourable views on gender-related political issues, suggesting political attitudes are motivated not only by one's own sex, but also the gender and reproductive opportunities of one's descendants. This may also help explain why women sometimes support policies that limit their autonomy and have the potential to be harmful to their personal wellbeing (see also Brooks & Blake, 2019). He et al. (2022), on the other hand, show that, for the Mosuo of China, while living with matrilineal kin may benefit women in some regards, the more reproducing women in the household the lower the probability is that the husband will help on a wife's farm. The authors suggest that decreases in a man's help on their wife's farm are motivated by potential fitness benefits of such help being diluted by unrelated members of their wife's household. Together these studies highlight the potential for evolutionary perspectives, by uniquely modelling fitness considerations, to provide novel insights into gender relations across and within cultural contexts.

## 3. Conclusion

We have sketched out the history of sexual/gendered conflict research in humans and proposed three priorities for future research. While we do not regard any proposition to be controversial, we also anticipate (and encourage) some healthy disagreement among scholars about the best means to meet each goal. A variety of actions are possible to diversify our scholarship, to modify conventional articulations of sexual conflict theory to incorporate the unique impacts of complex cumulative culture, and to effectively apply our observations to matters of policy concern. To this end, we conclude with some final reflections, and cautionary points, about the path forward.

First, with respect to diversifying the cultural background of researchers, we recognise that building a more internationally representative research community will take a number of actions beyond our approach to editing. In particular, institutional barriers remain fundamental, including inequitable access to funding streams, including indirect cost rates (i.e. the ability to charge institutional overheads) and limited national research budgets within LMICs (Haberer & Boum, 2023). Appropriate actions will vary by context, and are not limited to questions of authorship, which may be challenging to achieve in some circumstances (Urassa et al., 2021). We also caution that global research partnerships, even if defined by intellectual exchange, equitable authorship and grant sharing, can fail to foster research capacity. Instead they may reinforce dependency on external funding and promote networks through which talented LMIC scholars are recruited to work overseas, subsequently deprioritising independent research agendas and depleting local institutions (Ishengoma, 2011). Indeed, international research partnerships may not be the best answer, with larger gains to be derived from strengthening LMIC institutions in their own right (Sanganyado, 2021). Drawing from our own experience, we highlight the value of mentoring schemes, such as *AuthorAid* and the *Consortium for Advanced Research Training in Africa* (Somefun & Adebayo, 2020). We also encourage researchers to be attentive to how debates about these issues are playing out in parallel disciplines. Evolutionary social scientists are increasingly paying attention to fieldwork ethics (Broesch et al., 2020; McKerracher & Núñez-de la Mora, 2022), but may also have much to gain from further mirroring proactive movements within global health, such as shifts in editorial expectations regarding authorship, and grant regulations that incentivise local institutional and researcher involvement (Boum et al., 2018; Urassa et al., 2021).

Second, with regard to emphasising cultural determinants and expressions of *gendered conflict*, we reiterate that we are not attempting to deny the realities of biological differences between the sexes. We have little doubt that differing chromosomes, hormonal profiles and genitalia, and corresponding capacities for reproduction, have fundamental impacts on the behaviour of women and men. A focus on culture is also not mutually exclusive with the potential for sexual selection to have differential impacts on psychological adaptation. Nevertheless, cultural inheritance of gendered behaviour is undeniable, and a key distinguishing feature of what makes us human. Our call is simply to centralise acknowledgement of this difference to the study of human sex/gender roles, leaving the door open for alternative perspectives on exactly how best to integrate culture into evolutionary accounts of behavioural diversity (see Brown et al., 2011, Micheletti et al. 2022). Moreover, we encourage researchers to move away from combative narratives about the inherent superiority of evolutionary approaches to the ‘*Standard Social Science Model’* (Tooby & Cosmides, 1992), and instead reflect more optimistically on the potential gains of paying greater attention, and respect, to research developments in neighbouring fields where the social learning and performance of gendered behaviour and ideology have been more extensively studied.

Finally, with regard to policy recommendations, it is important to temper vaulting ambition by underscoring that additional skills and considerations are required in moving from purely academic concerns to more applied research. The promise of applied evolutionary social science has been the subject of several perspective pieces in recent years (Alvergne, n.d.; Gibson & Lawson, 2015; Jones et al., 2021; Schimmelpfennig & Muthukrishna, 2023; Tucker & Rende Taylor, 2007), including in reference to the COVID pandemic (Arnot et al., 2020), leading to many recommendations for how we can better communicate our findings to relevant stakeholders. We share this enthusiasm, but emphasise that caution is also needed when making policy recommendations, not least because poorly designed interventions have considerable potential to cause harm. We must critically reflect on the quality of our evidence and the likely generalisability of findings to heterogeneous contexts, and encourage effective use of piloting before making large-scale recommendations (IJzerman et al., 2020). More generally, evolutionary social scientists must keep informed of key critical debates within the global health literature, including for example, critiques of social norm approach to behaviour change (Wazir, 2023), evidence-based evaluation (Ravallion, 2020), the ‘harmful cultural practices’ framework (Winter et al., 2002) and associated tendencies for ‘culturalism’ (Pot, 2019), and the use of popular, but flawed measures of ‘development’ such as national income measures (Jerven, 2013) and common metrics of women's empowerment (Tavenner & Crane, 2022). Only by paying attention to these critical discussions, through our own training and collaborations outside of our field, can we expect to make meaningful contributions to tackling gendered conflict.

## Data Availability

n/a

## Research transparency and reproducibility

n/a
